# Impact of Dispersion Force Schemes on Liquid Systems: Comparing Efficiency and Drawbacks for Well-Targeted Test Cases

**DOI:** 10.3390/molecules27249034

**Published:** 2022-12-18

**Authors:** Evelyne Martin, Iréné Bérenger Amiehe Essomba, Kana Ishisone, Mauro Boero, Guido Ori, Carlo Massobrio

**Affiliations:** 1 Laboratoire ICube, Université de Strasbourg, CNRS, UMR 7357, F-67037 Strasbourg, France; 2 Institut de Physique et Chimie des Matériaux de Strasbourg, Université de Strasbourg, CNRS, F-67034 Strasbourg, France

**Keywords:** dispersion forces, first-principles molecular dynamics, chalcogenides, disordered networks, liquid and glassy systems, density functional theory

## Abstract

First-principles molecular dynamics (FPMD) calculations were performed on liquid GeSe_4_ with the aim of inferring the impact of dispersion (van der Waals, vdW) forces on the structural properties. Different expressions for the dispersion forces were employed, allowing us to draw conclusions on their performances in a comparative fashion. These results supersede previous FPMD calculations obtained in smaller systems and shorter time trajectories by providing data of unprecedented accuracy. We obtained a substantial agreement with experiments for the structure factor regardless of the vdW scheme employed. This objective was achieved by using (in addition to FPMD with no dispersion forces) a selection of vdW schemes available within density functional theory. The first two are due to Grimme, D2 and D3, and the third one is devised within the so-called maximally localized Wannier functions approach (MLWF). D3 results feature a sizeable disagreement in real space with D2 and MLWF in terms of the partial and total pair correlation functions as well as the coordination numbers. More strikingly, total and partial structure factors calculated with D3 exhibit an unexpected sharp increase at low k. This peculiarity goes along with large void regions within the network, standing for a phase separation of indecipherable physical meaning. In view of these findings, further evidence of unconventional structural properties found by employing D3 is presented by relying on results obtained for a complex ionic liquid supported on a solid surface. The novelty of our study is multifold: new, reliable FPMD data for a prototypical disordered network system, convincing agreement with experimental data and assessment of the impact of dispersion forces, with emphasis on the intriguing behavior of one specific recipe and the discovery of common structural features shared by drastically dissimilar physical systems when the D3 vdW scheme is employed.

## 1. Introduction

Disordered systems can play a benchmark role in assessing the sensitivity of structural properties to the inclusion of dispersion forces (van der Waals, vdW hereafter) within the Kohn–Sham formulation of the density functional theory (DFT) [1]. By focusing on the family of chalcogenides, it is worth starting by quoting the seminal paper devoted to liquid Ge_15_Te_85_, in which it was proposed that more realistic pair correlation functions and structure factors could be achieved by including vdW corrections [2]. Similar considerations were also developed later for liquid and amorphous GeTe [3,4]. However, it should be recognized that, despite their intrinsic validity, these contributions were more instrumental in opening a lively debate on the need of adding vdW corrections than in assessing their quantitative importance to improve the cohesive properties of disordered chalcogenides. For instance, some criticism on the general validity of the various dispersion force schemes was advanced in Ref. [5], stimulating a lively debate on the conceptual foundations underlying the inclusion of dispersion forces in DFT calculations. Advances in this direction were also obtained by our team, most significantly on liquid GeSe_2_ and glassy GeTe_4_, by using different vdW schemes within first-principles molecular dynamics (FPMD) [6,7,8,9]. We refer to Ref. [9] for a comprehensive account of what happens in the case of glassy GeTe_4_ when as many as four different strategies are employed to unravel the structural properties of this system under the effect of dispersion forces.

For the purpose of this paper, the theoretical frameworks leading to a treatment of vdW interactions can be listed as belonging to two distinct approaches. More precisely, the theoretical expression due to P. Silvestrelli [10,11,12,13] (making use of maximally localized Wannier functions, MLWF hereafter) is based on the update of the dispersion coefficients according to the time evolution of the electronic structure. This differs markedly from the so-called D2 and D3 recipes proposed by Grimme [14,15] featuring vdW contributions skillfully extracted from a large set of reference calculations but not sensitive to changes in the electronic structure during the dynamical evolution and, as such, not updated along a dynamical simulation.

A thorough study of the effect of dispersion forces on the structure of glassy GeTe_4_ revealed that the MLWF approach has optimal performances both in terms of comparison with experiments and realistic deviation from DFT structural data not including dispersion forces [9]. However, in a quite general fashion, the impact of vdW contribution on the atomic structure of disordered A_x_B_1−x_ chalcogenides remains moderate although not negligible, especially for small A_x_ concentrations. The question arises whether the indications previously reported for disordered chalcogenide systems also apply in the case of liquid GeS_4_, for which the only available FPMD data are limited to a system made of *n* = 120 atoms with only 24 Ge atoms [16]. Collecting new structural data on a larger system is by itself a valuable outcome, since the statistical accuracy of Ge-related properties cannot be firmly assessed for a periodic structure containing such a limited number of atoms for one species. In addition to this update on structural properties, obtained by employing a simulation cell of *n* = 645 atoms, we are also in a position to follow the behavior of various structural properties when different dispersion recipes are adopted, this novel and quite exhaustive result contributing to a precise understanding of the impact of dispersion forces on the structure of disordered chalcogenides. We stress that no FPMD data are available on this issue for liquid GeSe_4_ and, in general, there are only a few for analogous liquids. Most importantly, the account of several vdW schemes goes beyond the mere inclusion of an additional part within DFT, since it allows us to infer the advantages and limits of several approaches in a comparative fashion. Besides our previous work on glassy GeTe_4_, no results of this kind (i.e., several vdW schemes for a single disordered chalcogenide system) exist in the literature.

### 1.1. Motivation: Why Two Targeted Cases?

As stated in the introduction, our original motivation was to elucidate the role played by the dispersion contributions on disordered chalcogenides by focusing on liquid GeSe_4_. Moreover, this task has been accomplished for a much larger system than the one employed in previous calculations. However, the goals of this work have somewhat been extended due to the appearance of peculiar and totally unexpected structural features obtained for liquid GeSe_4_ when considering a specific vdW recipe, namely, D3. This prompted us to include in our FPMD computational effort a second disordered system, specifically an ionic liquid lying on a WSe_2_ substrate (see Section 4.1), for which vdW interactions are expected to be more important than in the case of liquid GeSe_4_ and contribute to the structure of the composite liquid–solid interface. This system exhibits structural anomalies (when using vdW-D3) sharing some similarities with liquid GeSe_4_, conferring on our findings a more general and instructive character. An account of this second system in the framework of a D3 calculation is the object of Section 4.1, to be considered as a complement to a study that remains largely devoted to liquid GeSe_4_. In view of the non-conventional atomic structures encountered for liquid GeSe_4_ with D3, we are convinced that such addition is of compelling importance to rationalize a phenomenon not limited to a single case.

Having specified the genesis and the evolution of our goals in conceiving this paper, it is worthwhile to point out that our primary purpose (FPMD calculations on liquid GeSe_4_ and sensitivity to the vdW forces) has been achieved by relying on three different theoretical schemes for the inclusion of vdW forces: D2, D3, and MLWF. One crucial issue, discussed in former papers [6,9], is their capacity not to alter artificially the basic structural organization (well known for being made of cross-chained GeSe_4_ tetrahedra connected to Se_n_ chains) while improving the agreement with experiments. There is no contradiction between introducing dispersion forces and expecting as a return only moderate changes in the structure. Indeed, vdW contributions have proved to be not essential to ensuring cohesion in disordered chalcogenides and yet their contribution can be non-negligible.

### 1.2. Focusing on Liquid GeSe_4_ as Main Structural Target: Reviewing Previous Results

Back in 1998, GeSe_4_ was one of the first non-oxide systems selected for a study of structural properties in disordered networks by FPMD [16]. In that study, the purpose was to describe at the atomic scale a liquid chalcogenide featuring Se atoms arranged in two distinct motifs, GeSe_4_ tetrahedra and Se_n_ chains. The different nature of chemical bonding for Se atoms in these configurations is predominantly ionic, due to GeSe_4_ tetrahedra, or covalent, as arising from Se_n_ chains. This composite scenario called for the use of FPMD as a predictive approach to be preferred to classical molecular dynamics based on interatomic potentials. Within the Ge_x_Se_1−x_ family of systems, the concentration x = 0.2 emerged as worth dwelling on, since it marks the boundary between two elastic behaviors well known within phenomenological pictures. The existence of this boundary stems from the equality between the number of constraints and the number of degrees of freedom, and it was later reconsidered by introducing the notion of intermediate phase and structural variability [17,18,19].

On the side of FPMD, intense efforts have been deployed since to describe the glassy structure for x = 0.2 in various kinds of chalcogenides, GeSe_4_ being highly representative because of the availability of experimental data. For instance, two system sizes (*n* = 120 and *n* = 480) were used for a comparative study between GeSe_4_ and GeS_4_ [20]. Worth mentioning are also the structural analyses of GeSe_4_ and SiSe_4_ in connection with the notion of intermediate phase [21] as well as the account of several exchange–correlation functionals in the case of GeSe_4_ reported in Ref. [22]. Overall, the absence of phase separation between GeSe_4_ and Se_n_ subnetworks [23,24] stands as an important outcome of FPMD calculations on glassy GeSe_4_.

Here, we have produced FPMD trajectories for a system much larger than the one used in 1998 (*n* = 645 atoms) by obtaining robust time averages for the macroscopic properties. It should be noted that several GeSe_4_ liquids were produced in the past at glass densities as initial steps of thermal cycles targeting the glassy phase [20]. However, as such, they were never exploited to obtain information on the corresponding atomic structure. This paper fills this gap by showing that liquid GeSe_4_ is made of a predominant number of corner-sharing connections in which Ge atoms are mostly fourfold coordinated with Se atoms, even though there are also about 10 per cent of them that have 3 Ge and one Se atoms in the first shell of coordination. The Se subnetwork is composed of a large majority of twofold-coordinated Se, three coordination motifs being observed: Ge-Se-Ge, Ge-Se-Se, and Se-Se-Se. By leaving aside the specific case of the vdW recipe D3 [15], the results showed little sensitivity to the dispersion scheme employed, legitimating the use of a FPMD prescription that does not include any dispersion correction.

## 2. Liquid GeSe_4_ by FPMD: Calculation Methodology

Our calculations are based on the Car–Parrinello FPMD methodology (CPMD, implemented in the corresponding CPMD code [25]) that we have extensively employed for chalcogenide glasses since the late nineties. For a collection of examples and detailed information on the methodological ingredients of FPMD applied to amorphous/glassy structures, see Ref. [26].

**Figure 1:**
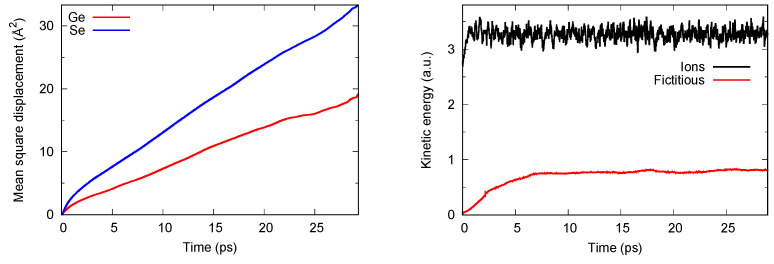
**Left side**: behavior of the mean square displacement for liquid GeSe_4_ at T = 1073 K. **Right side**: total kinetic energy for the ionic (**top part**) degree of freedom and the fictitious electronic degrees of freedom (**bottom**). Without loss of generality, we are referring here to FPMD calculations performed within the MLWF scheme for the dispersion forces.

The system is made of 645 atoms (N_Ge_ = 129, N_Se_ = 516) in a cubic periodic box with edge 28.03 Å to reproduce the experimental density [27]. The initial coordinates were extracted from previous simulations on disordered chalcogenides and adequately randomized to lose memory of the initial conditions. DFT was implemented within the FPMD scheme by adopting BLYP as the generalized gradient correction (GGA). BLYP consists of the exchange functional by Becke [28] and the correlation functional by Lee et al. [29]. This choice is well suited for disordered chalcogenides since it increases electronic localization, thereby remedying drawbacks found when the uniform electron gas is taken as the reference system. As customarily used in CPMD applications, we employed a plane-waves basis set, for which convergence is ensured in a range of representative properties (interatomic distances, frequencies, cohesive energy) with an energy cutoff of 40 Ry (544.23 eV). The Brillouin zone integration is restricted to the Γ point, as appropriate for insulator or semi-conductor disordered systems. Thermostats on the ionic (**R**_i_) and fictitious electronic degrees of freedom φ*_l_* (the index *l* running on all electronic eigenstates) were implemented via the Nosé–Hoover scheme [30] extended to the electronic wavefunctions by Blöchl and Parrinello [31], allowing for efficient separation of these two families of degrees of freedom. In this way, adiabaticity is ensured, i.e., no transfer of energy between **R**_i_ and φ*_l_*, with φ*_l_* lying dynamically close to the Born–Oppenheimer surface. An example of such realization is given in Figure 1. The fictitious mass associated to φ*_l_* is equal to µ = 1000 a.u. Values for the masses of the thermostats (in units of corresponding frequencies) are taken equal to 300 cm^−1^ for the ions and in the range 500–700 cm^−1^ for the fictitious electronic degrees of freedom.

Prior to the CPMD runs bringing the system to a high temperature, the initial configuration is relaxed to a structural local minimum. This allows extracting the residual thermal energy by providing an atomic arrangement characterized by negligible forces with the corresponding electronic structure very close to the ground state. For CPMD simulations, the realization of such initial “time 0 configuration” (T0C) is an unavoidable prerequisite before moving to finite temperature simulations. Each one of the four dispersion schemes applied within DFT-FPMD (no inclusion of vdW effects, termed No-vdW, D2, D3, and MLWF; see below for further details) starts from T0C with runs of equal lengths at the different temperatures. These are carried out by implementing a thermal cycle with the ionic thermostat bringing the system in sequential steps to T = 300 K, T = 600 K, T = 900 K, and T = 1073 K, each step lasting at least 30 ps.

The reliable realization of the CPMD scheme and the significance of the phase space sampling can be recognized by monitoring relevant quantities such as temperatures (ionic and fictitious) and mean square displacements, as shown in Figure 1. Two features are worthy of note. Both Ge and Se atoms cover distances corresponding to several interatomic spacings, pointing out the effective randomization and loss of any memory of the initial configuration. Moreover, the instantaneous temperature (kinetic energy) associated with the ionic degrees of freedom **R**_i_ is well above that of the fictitious electronic degrees of freedom φ*_l_*, thereby ensuring adiabaticity. The results presented in what follows have been obtained as averages over at least 20 ps at T = 1073 K. For reasons that will be commented upon in Section 3 and Section 4, a second trajectory was produced for the D3 case starting from the end of the D2 one and lasting 30 ps. The results for the two D3 trajectories will be clearly identified by different colors in the figures and by the labeling of D3(A) and D3(B) in the tables.

### Dispersion Forces within First-Principles Molecular Dynamics

In this section, we shall briefly review some basic notions concerning the treatment of dispersion forces within DFT-based FPMD methods. Here, we consider uniquely the case of dispersion effects treated as a quantity separable from the main body of the Kohn–Sham Hamiltonian and linearly added to it, adopting the expression *C*_6_$/r_{ij}^{6}$, with $r_{ij}$ the interatomic distances between a pair of atoms *i* and *j*. By following S. Grimme and his original formulation termed “D2” [14], the dispersion contribution to be added to the total energy takes the form

(1) $$\;\;\;\;E_{vdW}^{D2}=-s_{6}\overset{Natoms}{\underset{i,j>i}{\sum }}\frac{C_{6}^{ij}}{r_{ij}^{6}}f_{damp}\left(r_{ij}\right)$$

where $C_{6}^{ij}$ is the coefficient for the atom pair *i* and *j* parameterized by quantum chemical calculations performed purposely on a wide set of training systems, $s_{6}\;$is a scaling factor depending on the type of exchange–correlation functional used in these calculations, and $f_{damp}\;$is a suitable damping coefficient. We have employed the D2 methodology to understand the impact of dispersion forces on chalcogenides for the cases of glassy GeTe_4_ [7,8,9], liquid GeSe_2_ [6], glassy GeSe_4_, glassy GeS_4_ [32], and glassy Ga_10_Ge_15_Te_75_ [33]. Along the lines pioneered with D2, Grimme and coworkers later proposed a refinement, named “D3”, including also an additional energy correction complementing the formula in Equation (1), termed E^(3)^, and consisting of a three-body interaction as derived from the third-order perturbation theory [15]. This extended vdW correction has been pointed out as affected by “less empiricism”. D3 aims not only at being used in connection with several exchange–correlation functionals but also features improvements in terms of cost and robustness. These ideas are claimed to provide unprecedented performances for the treatment of dispersion forces. However, a common feature of both the D2 and the D3 schemes is that they are not intended to account for on-the-fly changes in the electronic structure with time. D3 calculations have been performed for glassy GeTe_4_ [9]. We found D3 in better agreement with experiments than D2, confirming the prediction of its less empirical character.

In addition to D2 and D3, in this study we also pursued the application of an alternative recipe for the dispersion forces due to P. L. Silvestrelli [11,12]. This approach makes use of the maximally localized Wannier functions (MLWF) $w_{n}\left(r\right)$ [10]. Accordingly, the coefficients $C_{6}^{ij}$ of Equation (1) take the following form (with indices *n*, *l* at the place of *i j*):

(2) $$C_{6}^{nl}=\frac{3}{32\pi^{3/2}}\int_{\left|r\right|<r_{c}}^{}d^{3}r\int_{\left|r^{\prime }\right|<r_{c}}^{}d^{3}r^{\prime }\frac{w_{n}\left(r\right)w_{l}\left(r^{\prime }\right)}{w_{n}\left(r\right)+w_{l}\left(r^{\prime }\right)}$$

The advantage of Equation (2) is its dependence on the localized character of $w_{n}\left(r\right)\;$since, in principle, the expression based on the local electronic density $\rho_{n}\left(r\right)$ is written as

(3) $$C_{6}^{nl}=\frac{3}{32\pi^{3/2}}\int_{\left|r\right|<r_{c}}^{}d^{3}r\int_{\left|r^{\prime }\right|<r_{c}}^{}d^{3}r^{\prime }\frac{\sqrt{\rho_{n}\left(r\right)\rho_{l}\left(r^{\prime }\right)}}{\sqrt{\rho_{n}\left(r\right)}+\sqrt{\rho_{l}\left(r^{\prime }\right)}}$$

but it is affected by the lack of a rigorous *local* definition for the total electronic density, particularly when non-localized basis sets, such as plane waves, do not allow for an unbiased partitioning of the density. Equation (2) is the appropriate tool to include information on the electronic structure in the dispersion coefficients without the need to go through more cumbersome treatments of the long-range interactions. An additional simplification in handling the MLWF is that the electronic structure information can be reduced to four numbers, the position (*x*,*y*,*z*) of the MLWF center of mass (Wannier function center, WFC), and its spread, which allow for an analytical formulation of the functions $w_{n}\left(r\right)$ and thus an easy implementation of Equation (2) [11,12,13]. We stress that this implementation based on localized orbitals allows following small electronic effects due to changes in the atomic configurations, readily affecting the values taken by the dispersion coefficients. The MLWF approach has been applied to the case of liquid GeSe_2_ [6], glassy GeTe_4_ [8], glassy GeSe_4_, and glassy GeS_4_ [32] in the framework of a comparative analysis with the D2 and D3 (GeTe_4_ only) methodologies. In the case of GeTe_4_, MLWF was found to be a good choice by limiting artifacts when the changes due to dispersion forces are expected to be small.

## 3. Liquid GeSe_4_: Results

In the present section, we obtain new benchmark reference data for liquid GeSe_4_ by exploiting a larger system (*n* = 645 atoms). In this way, our results have a much higher statistical accuracy when compared to those of Ref. [16] obtained with *n* = 120. Moreover, in view of the agreement obtained in Ref. [16] between FPMD results and available experimental data, we organize this section by pursuing a comparison among the different dispersion schemes, with the scope of understanding how different formulations of the vdW interaction can impact the corresponding structural properties. Results in real space are presented first, followed by results in reciprocal space.

### 3.1. Real-Space Results

**Figure 2:**
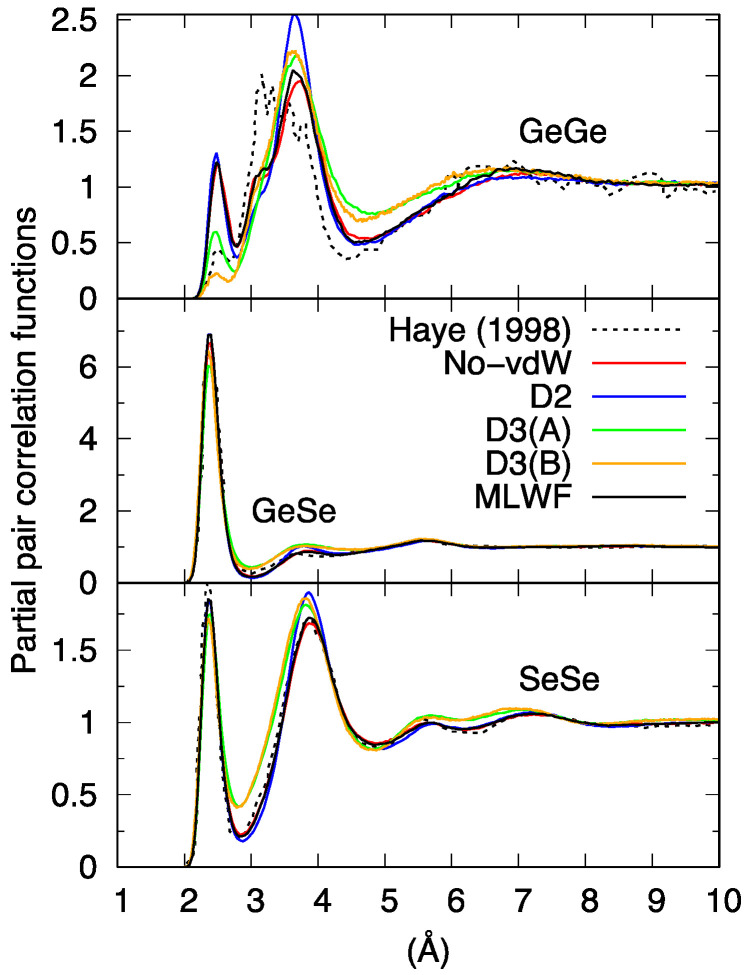
Calculated partial pair correlation functions for liquid GeSe_4_. Haye (1998) stands for Ref. [16].

Figure 2 shows the partial pair correlation functions g_Ge-Ge_(r), g_Ge-Se_(r), and g_Se-Se_(r) calculated with the different vdW schemes, the comparison including the No-vdW results of Ref. [16] (*n* = 120) and the new set of data produced in the present study (*n* = 645). As a first consideration, and leaving aside for the moment the D3 results, it appears that g_Ge-Se_(r) and g_Se-Se_(r) show very little sensitivity to the presence of dispersion forces, exhibiting very similar peak positions and intensities. Moreover, g_Ge-Se_(r) and g_Se-Se_(r) are not markedly affected by the larger size of the system, showing that the basic features of the network (GeSe_4_ tetrahedra and Se_n_ chains) were already sufficiently well described in the *n* = 108 model. These statements are supported by the analysis presented in Table 1, containing information on the network topology as obtained via the partial coordination numbers relative to the interaction of a given atom with neighboring atoms of the same or a different species ($n_{Ge}^{Ge}$ and $n_{Ge}^{Se}$ for *Ge*, $n_{Se}^{Ge}$ and $n_{Se}^{Se}$ for *Se*). Table 1 also provides the total coordination number of a given species resulting from the sum of the above partial coordination numbers $n_{Ge}^{tot}=n_{Ge}^{Ge}+n_{Ge}^{Se}\;$ and $n_{Se}^{tot}=n_{Se}^{Ge}+n_{Se}^{Se}$.

The situation changes drastically when focusing on g_Ge-Ge_(r) and the corresponding coordination numbers. Visual inspection of Figure 2 reveals that the new set of No-vdW data is much less noisy than the one obtained in 1998, exhibiting a larger number of corner-sharing (CS) connections (peak around 3.6–3.8 Å) and only a small shoulder at interatomic distances typical of edge-sharing (ES) connections (around 3.2 Å). While the analysis of Ref. [16] points toward very close numbers of CS and ES connections (49% of Ge atoms part of fourfold rings were reported in Ref. [16]), we found only 20% of Ge atoms in ES motifs, showing that a more extended statistical sampling has a remarkable effect on the minority species Ge. Another interesting feature associated with the new No-vdW results is the presence of a discernible number of homopolar Ge-Ge bonds, leading to coordination numbers close to 0.2, four times more than in Ref. [16], where this quantity was not explicitly reported. This result is in line with the presence of Ge atoms that do not form tetrahedral connections with Se atoms. Focusing on the comparison among g_Ge-Ge_(r) pair correlation functions for different dispersion recipes, the profiles for the No-vdW, D2, and MLWF results are globally close, a higher peak at ~3.8 Å being recorded for D2. Variations are also minimal when looking at the coordination numbers of Table 1.

When compared to the cases of No-vdW, D2, and MLWF, the results corresponding to the application of D3 are peculiar for all partial pair correlation functions, although the differences noticeable in the g_Ge-Se_(r) case are less striking (a less intense first peak and first minimum, together with an overall flatter shape for the profile at larger distances). For all schemes, the coordination numbers $\;n_{B}^{A}\;$ relative to the Ge-Se mutual environments are close to the values pertaining to a chemically ordered network, $n_{Ge}^{Se}=4$ and $n_{Se}^{Ge}=1\;$ but the deviations are larger for the D3 case: $n_{Ge}^{Se}=3.56\;$ and $n_{Se}^{Ge}=1.18$. Anomalies in the behavior of D3 appear also in the pattern of g_Se-Se_(r), characterized by a less profound first minimum, whereas No-vdW, MLWF, and D2 are nearly identical, this latter differing only by the height of the second peak.

**Figure 3:**
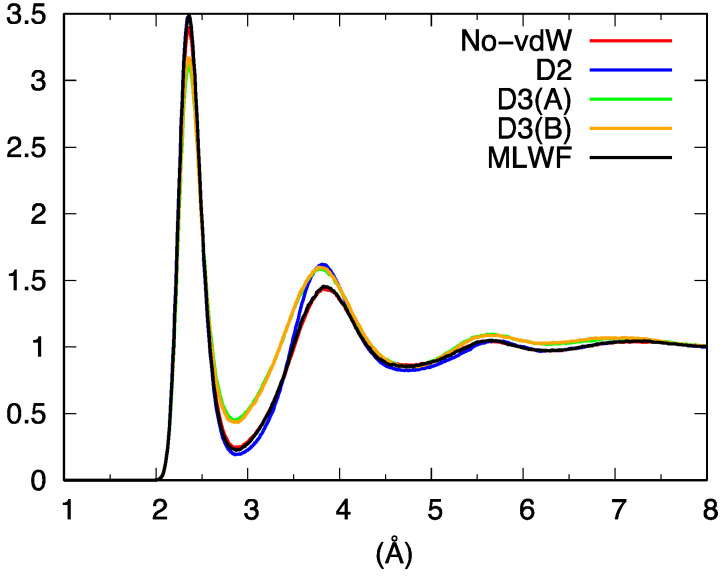
Total pair correlation function for liquid GeSe_4_.

Overall, the picture arising from this first set of comparisons involving our four distinct FPMD calculations can be summarized by noting that D3 behave differently from No-vdW, D2, and MLWF, the resemblance of these three approaches being a first indication of the low sensitivity of liquid GeSe_4_ to the inclusion of dispersion forces. The total pair correlation function (Figure 3) reflects these results, exhibiting a less pronounced profile at short distances for D3 and differences among No-vdW, MLWF, and D2 limited to the height of the second peak.

**Figure 4:**
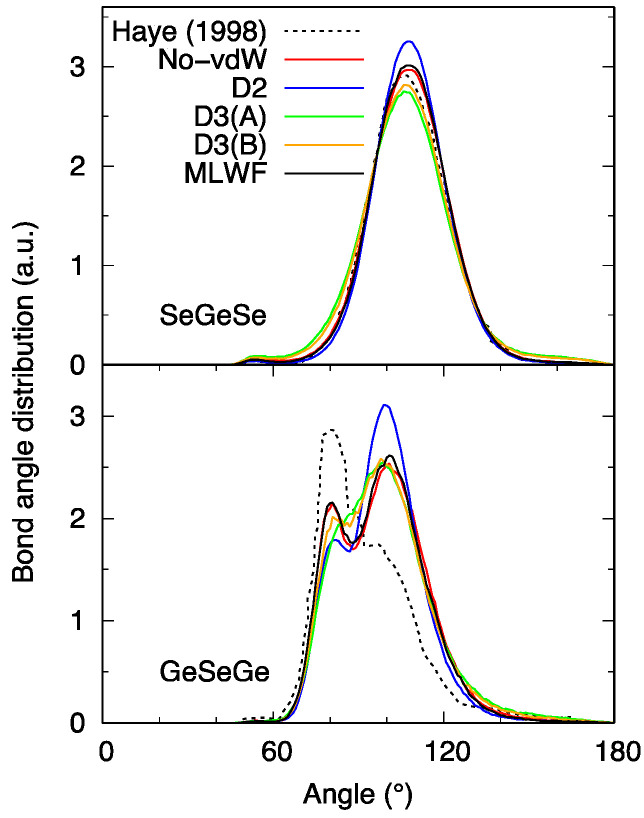
Bond-angle distribution functions (BAD) for liquid GeSe_4_. Haye (1998) stands for Ref. [16].

The network topology can also be described in terms of the bond-angle distributions (BAD) (Figure 4) indicating a predominant tetrahedral arrangement within the Ge_x_Se_1−x_ family. In the Se-Ge-Se case, one recognizes the peak around 108° due to the inter-tetrahedron Se-Ge-Se connections. Variations in the intensity amount to 10% at most, with the highest and the lowest values corresponding to D2 and D3, respectively.

The Ge-Se-Ge BAD is more insightful in capturing the changes occurring when considering the present *n* = 645 system (No-vdW results) in comparison to the results obtained with the smaller model of Ref. [16] obtained with short time trajectories. As expected on the basis of the shape of the pair correlation functions, we do have a profile with a second peak, due to CS connections, higher than the first one, resulting from ES connections. The correspondence between the kind of connectivity and the range of angles has been firmly established in the past for other Ge-Se systems [26] and can be exploited here to conclude that CS connections are predominant, the majority of ES linkages found in Ref. [16] arising from insufficient statistical sampling. D3 calculations are more intriguing in this context, since the double-peak structure is not clearly defined, as if the network were lacking a regular pattern of well-distinct CS and ES connections.

As a final piece of information on the network structure, Table 2 and Table 3 provide the individual structural units, giving the percentages of Ge or Se atoms onefold coordinated to other atoms. For instance, Ge-GeSe_3_ stands for a Ge atom connected to four neighbors, three Se atoms and one Ge atom; GeSe_4_ means a Ge atom connected to four Se atoms; and Se-GeSe_2_ stands for one Se atom with one Ge atom and two Se atoms as nearest neighbors. In Table 2, more than 80% of Ge atoms are found in fourfold coordination for No-vdW, D2, and MLWF calculations, either with four Se atoms or with one or two Ge atoms and three or two Se atoms. This is consistent with a sort of enhanced dynamical disorder disrupting a chemically ordered tetrahedral network, bound to be restored upon annealing at lower temperatures as observed for glassy GeSe_4_ and GeS_4_ [20]. Threefold Ge atoms are a minority accounting for less than 10%. Along the same lines, Table 3 provides evidence for twofold connections being the most frequent in the Se case, distributed among Se-Se-Se, Se-Se-Ge, and Ge-Se-Ge connections. However, while No-vdW, D2, and MLWF calculations give as many as ~88% of twofold-coordinated Se atoms, these percentages reduce by about 10% in the D3 case.

We can summarize the analysis of the structure in real space by stating that longer runs and a larger system (improving upon the statistics of Ref. [16]) modify the relative number of ES and CS connections in favor of these latter. Moreover, the inclusion of dispersion forces does not appear to alter significantly the structural properties when referring to the D2 and MLWF approaches. More puzzling is the behavior of the pair correlation functions, bond angular distribution, and the counting of structural units resulting from the use of the D3 scheme. The atomic structure in this case departs from a network made of Ge tetrahedrally connected and Se either connected in tetrahedra or arranged in chains. These basic motifs persist and yet they are less predominant as if extra contributions to chemical bonding had a stronger influence. Given these conclusions and the availability of experimental data [27] in reciprocal space (the total neutron structure factor S_tot_(k) employed in Ref. [16] to substantiate our previous FPMD calculations), a comparison of all results for S_tot_(k) is particularly insightful.

### 3.2. Reciprocal-Space Results

**Figure 5:**
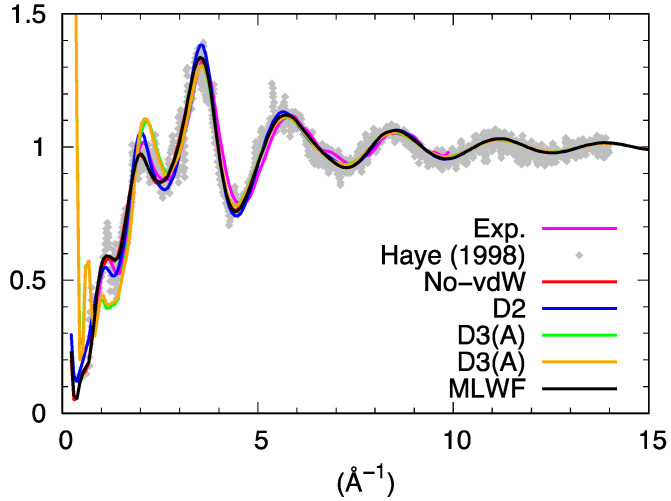
Total neutron structure factor of liquid GeSe_4_. The experimental data are those of Ref. [27]. Haye (1998) stands for Ref. [16].

The total neutron structure factor S_tot_(k) of liquid GeSe_4_, obtained by linear combination of the partial structure factor by accounting for the coherent neutron scattering lengths and the concentration of the two species is shown in Figure 5. For the partial structure factors, we provide the result corresponding to the integration from real space of the pair correlation function up to the largest available range. This result was found to be less affected by statistical noise than the direct calculations in reciprocal space, as detailed in the literature [26]. The essence of the conclusions presented hereafter are by no means depending on this choice, mostly adopted for the sake of clarity. S_tot_(k) resulting from the various dispersion schemes agrees well with the previous results of Ref. [16] and the experimental data of Ref. [27] provided one refers to the range of values of k larger than 2 Å^−1^ in reciprocal space.

**Figure 6:**
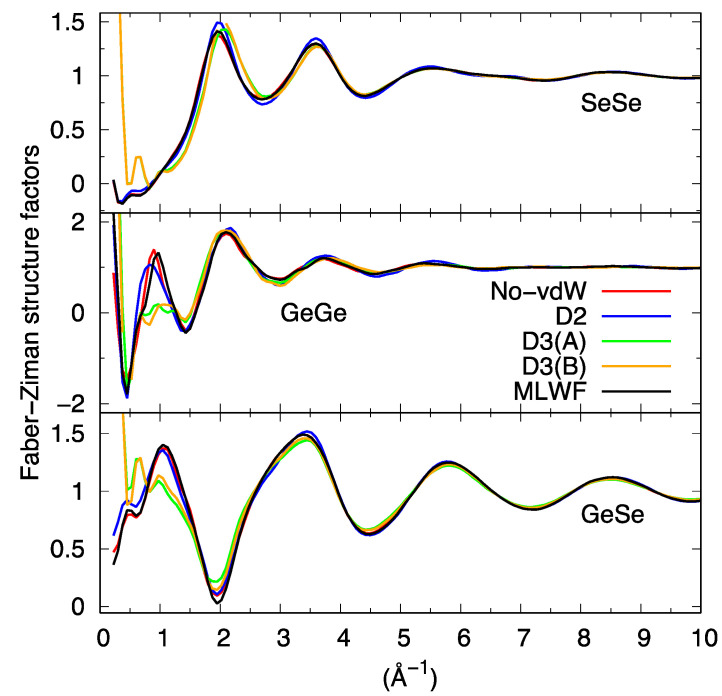
Partial structure factors for liquid GeSe_4_ obtained via integration of the partial pair correlation functions.

However, two notable exceptions can be remarked at lower k. The first one is the strong disagreement recorded around 1 Å^−1^ when using D3 (for both D3(A) and D3(B) trajectories) as if the intermediate range order typically manifesting itself via the presence of a peak around 1 Å^−1^ were absent. The second, more surprising, is the appearance of an outstanding feature detectable at very low values of k in the D3 cases. The profile of S_tot_(k) moves sharply upwards for k going to zero, as also clearly recognizable via the decomposition in terms of the partial structure factors (Figure 6).

While some kind of unexpected increase at k~0.2–0.4 Å^−1^ is also present for S_Ge-Ge_(k) in the MLWF and D2 cases, indicating a form of unforeseen correlations involving the Ge subnetwork, D3 is the only dispersion scheme exhibiting this pattern in the three partial structure factors S_Ge-Ge_(k), S_Ge-Se_(k), and S_Se-Se_(k). For the D3 case, such a sharp increase common to the three partials is at the very origin of its appearance in S_tot_(k), since the weight of S_Ge-Ge_(k) is the smallest among the three and has a vanishing impact for No-vdW, D2, and MLWF calculations.

It is appropriate at this point to legitimate *a posteriori* the production of a second D3 trajectory (D3(B) as anticipated in the methodology section) to prove or disprove the indications of the first and wipe out any ambiguities that could arise from conclusions based on a single trajectory. Globally, D3(B) gave the same results as D3(A) (see Figure 5 and Figure 6). Based on these pieces of evidence, our first conclusions indicate rather clearly a quite peculiar and intriguing behavior characterizing all atomic correlations (Ge-Ge, Se-Se, and Ge-Se) for D3 when analyzed in reciprocal space, contributing to what can be observed in Figure 5 for S_tot_(k).

## 4. Discussion

**Figure 7:**
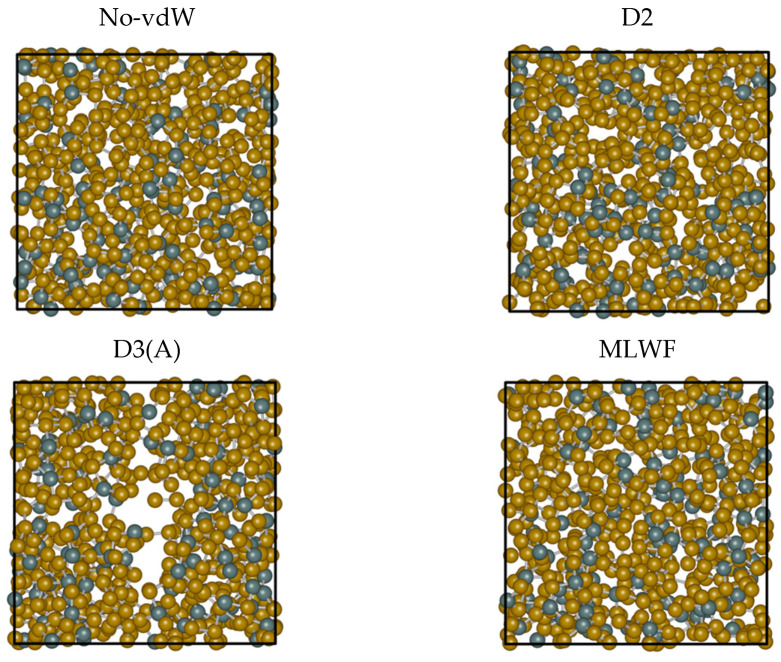
Snapshots of configurations obtained for liquid GeSe_4_ at T = 1073 K by using the four different treatments for the inclusion of the dispersion forces detailed in the text. From top to bottom and from left to right: No-vdW (**top left**), D2 (**top right**), D3 ((**bottom left**), first trajectory), MLWF (**bottom right**). The snapshots were produced by iRASPA [34]. The Ge atoms are in dark green, the Se atoms in brown, and the unit cell is represented with a black line.

**Figure 8:**
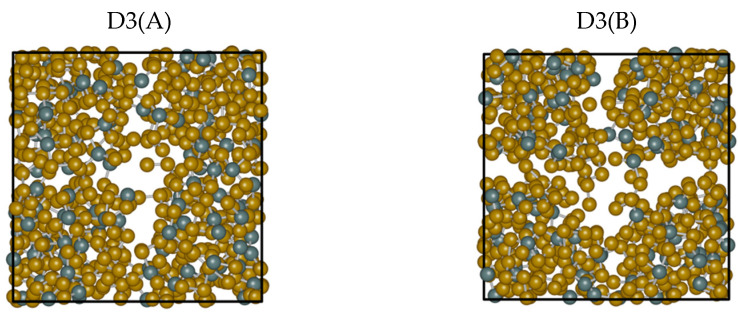
Snapshots of configurations obtained for liquid GeSe_4_ at T = 1073 K by using two trajectories for the D3 case, D3(A), and D3(B), the second being produced from the final state of D2. **Left side**: snapshot extracted from the D3(A) trajectory. **Right**: snapshot extracted from the D3(B) trajectory. The snapshots were produced by iRASPA [34]. The Ge atoms are in dark green, the Se atoms in brown, and the unit cell is represented with a black line.

A direct visual inspection of the structures obtained for the different dispersion recipes reveals some macroscopic effects consistent with the peculiarity exhibited by D3. Figure 7 is insightful in this respect. While the snapshots extracted from No-vdW, D2, and MLWF trajectories exhibit a fully homogenous arrangement in space, the snapshot relative to D3 is indicative of an inner phase separation. This phenomenon, in addition to being difficult to grasp on physical grounds, bears no resemblance to the phase separation between GeSe_4_ and Se_n_ subnetworks hypothesized in the past and is not substantiated by any FPMD calculations [21,22,23,24]. The puzzling evolution of the trajectory generated via D3(A) is confirmed when starting from a different initial configuration (final configuration of the D2 trajectory) and performing an additional 30 ps trajectory (D3(B) results). A comparison of the two topologies is provided in Figure 8. The second D3 trajectory, generated from an entirely different initial configuration (D2), witnesses the persistence of and the increase in a large region inside the system essentially devoid of atoms. Overall, this topology gives rise to correlations extending over a large part of the network over distances exceeding any conceivable long-range interaction. This feature is at the very origin of the sharp increase observed for S_tot_(k) for k approaching zero.

The question arises whether or not the behavior recorded with D3 bears some realistic character or it stands for a new phenomenon either unpredictable without considering dispersion forces or not detectable when adopting D2 or MLWF. In view of our results showing the worst agreement on both real-space and reciprocal-space properties for D3, we tend to conclude that the voids appearing when D3 is employed are mere artifacts of that scheme. In what follows, by relying on a further example of shortcomings occurring when using D3 for a totally different system, we shall try to determine the origin of such unexpected behavior, with the only intent of contributing to a better control of these important theoretical tools.

### 4.1. The Case of an Ionic Liquid Interacting with A Solid Substrate

The performances of the same vdW formulations (D2, D3, and MLWF) were tested in a different context on a typical system composed of an ionic liquid (IL) in contact with a solid substrate. This type of system is being pioneered as a promising major component in 2D-material-based field-effect transistors [35,36,37]. It has the advantage of operating as a gate able to induce a remarkably high interface charge which, in turn, reduces the operating voltages, thereby minimizing the energy consumption [35,36,37].

Our FPMD study focused on the ionic liquid (IL) 1-Ethyl-3-methylimidazolium bis(trifluoromethylsulfonyl)imide, hereafter indicated with the standard acronym [EMIM] [TFSI] ([EMIM] being the cation and [TFSI] the anion), on a WSe_2_ substrate [37,38]. A thorough FPMD study, accompanied by an extensive benchmark toward experimental data, has been reported elsewhere for the [EMIM] [TFSI] system [39]. On that occasion, a MLWF scheme was used to simulate the IL, obtaining a remarkable agreement with all available experimental data. This ionic liquid was then put in contact with a WSe_2_ substrate consisting of two layers of material. This type of solid support consists of a stacking of WSe_2_ layers held together by vdW forces only, whereas the IL is prone to promote a variety of interactions with the substrate (electrostatic interactions and hydrogen bonding) and is a major experimental research target [37,38,40,41,42], although still at the pioneering stage on a computational standpoint [43,44]. In our specific case, for this composed system of relatively large size, we use a model made of two WSe_2_ layers in which the bottom one is fixed to the bulk crystallographic coordinates, whereas the upper layer is allowed to follow dynamically the evolution of the unconstrained [EMIM] [TFSI] liquid on top of it. Such a system consists of 20 [EMIM] [TFSI] ionic pairs (680 atoms) plus the 150 atoms (50 W and 100 Se) of the substrate, for a total amount of 830 atoms in a hexagonal cell of 17.07 × 17.07 × 65.0 Å^3^, corresponding to a 5 × 5 replica of the unit cell of WSe_2_. To preserve the stacking of the layers in the WSe_2_ substrate, on the corner atoms of the upper layer, a dynamical restraint of the type *V*_res_ = *k* (**R**(*t*) − **R**_0_)^2^ was imposed.

**Figure 9:**
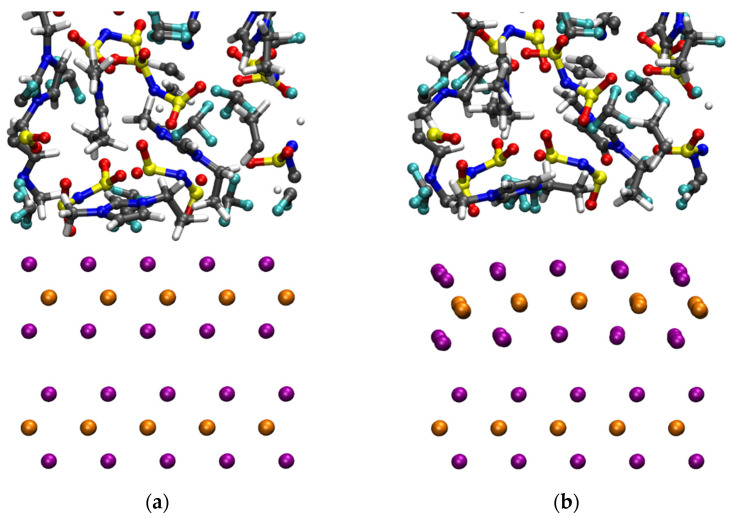
Snapshots of configurations of the ionic liquid [EMIM] [TFSI] obtained at T = 300 K by using the vdW scheme D2 (or MLWF, panel (**a**)) and D3 (panel (**b**)) within the same FPMD framework. In the figures, the orange and purple spheres in the bottom layers represent the W and the Se atoms, respectively. For the ionic liquid, the atom color code is gray for C, blue for N, red for O, yellow for S, cyan for F, and white for H.

In addition to the MLWF scheme of the original IL work [39], we have considered D2 and D3 vdW corrections in an attempt at reducing the computational cost while preserving the accuracy. For the purpose of the present paper, we shall focus exclusively on the results obtained via NVT dynamics with a constant volume and temperature within the D3 approach in comparison with a D2 or a MLWF one. These results are summarized in Figure 9. A visual inspection of the structure realized by the different vdW prescriptions reveals an anomalous effect detected only when the D3 scheme is used. This consists in an unexpected curvature of the upper WSe_2_ layer, i.e., the one evolving dynamically and not fixed to crystallographic positions. After about 3 ps of dynamics in the canonical NVT ensemble at 300 K, within the D3 scheme, the two layers are being held together uniquely by the restraint *V*_res_ imposed on the corner atoms. The vdW-D3 acting among the atoms of the first and second layers tend to separate these two layers instead of keeping them packed, as is well known for this specific solid in which the vdW interaction is a dominant feature [37,38]. The interatomic distance between the Se atoms facing each other in the two layers, Se_layer1-top_-Se_layer2-bottom_, reaches ~3.0 Å for the central atoms away from the restraints.

This has to be compared with a regular 2.75 Å close to the experimental value and reproduced by D2 or MLWF dispersion corrections. This configuration is indicative of a sort of repulsion, opposed to what a vdW interaction is expected to produce. At the same time, the IL shows a clear trend of departure from the WSe_2_ surface, breaking all the H-bonds obtained after D2 equilibration, resulting in an artificial phase separation between the IL and its solid support. The liquid is separated from it by distances larger than 3.0–3.5 Å, as is typical of H-bonding characterizing this system [38,39].

This anomalous behavior is consistent with the strongly non-homogeneous density phenomenon highlighted in the chalcogenides system of Section 3. Is it possible to ascribe these peculiarities to some specific part of the D3 analytical expression? By coming back to the expression of the various vdW formulations considered here, the only major feature proper to D3 and absent in D2 and MLWF is the E^(3)^ term present in D3, whereas all the other formulae reduce basically to the same term consisting of the *C*_6_ coefficient divided by r^6^. It is then very much unlikely that this part of the vdW interaction, a common feature of all schemes employed here, can behave differently in the D3 scheme. Therefore, we can advance the conjecture that the intriguing behaviors reported above for two cases (a disordered chalcogenide and a supported ionic liquid) are due to the three-body term inherent in D3. Despite the evidence collected on drawbacks related to the use of D3, our results have to be taken as a simple warning not intended to undermine the theoretical foundations of D3 but merely calling for more extensive studies to understand the role of this specific scheme in other disordered systems.

## 5. Conclusions

The original purpose of this paper was twofold: (*a)* the production of new FPMD data on liquid GeSe_4_, superseding those of Ref. [16] obtained in 1998 on a system made of 120 atoms only and on relatively short time trajectories, and (*b)* the analysis of the sensitivity of the atomic structure to different schemes for the treatment of the dispersion forces.

The most relevant results obtained are as follows:

(1) Globally, the inclusion of dispersion forces does not alter the basic structural features of liquid GeSe_4_ that can be described as a network in which GeSe_4_ tetrahedra coexist with Se_n_ chains.
(2) On the quantitative point of view, considerations of a larger size allowed recovering a higher percentage of corner-sharing tetrahedra, in line with previous results collected for other disordered chalcogenide systems [26].
(3) We found that some notable differences appear in the case of D3 when considering the partial and total pair correlation functions and the bond angle distributions, as if the tetrahedral order typical of the GeSe_4_ network were somewhat altered.
(4) These observations are confirmed by reciprocal space data and a comparison with the experimental counterpart revealing a striking, unexpected feature (i.e., an increasing spike) at very low wavevectors in the D3 case. Snapshots of the corresponding structure feature a phase separation taking place within the network in which void regions are clearly noticeable.
(5) In view of these findings, we have included in this paper a second example of intriguing structural behavior that arises when employing the D3 dispersion scheme (a complex ionic liquid supported by a WSe_2_ layer). The action of a three-body force built in D3 is likely to be at the origin of a possible unphysical contribution.

Overall, we do hope our findings will foster further thoughts on the role of dispersion forces and their implementation within DFT, so as to contribute to a general understanding of the relationship between physical properties and this kind of week interaction, as shown in recent accomplishments [45].

## Figures and Tables

**Table 1:** Coordination numbers of liquid GeSe_4_ calculated with the various schemes for the dispersion forces detailed in the text. The values have been obtained by integrating the partial pair correlation functions up to the first minima.

	$n_{Ge}^{Ge}$	$n_{Ge}^{Se}$	$n_{Ge}^{tot}$	$n_{Se}^{Se}$	$n_{Se}^{Ge}$	$n_{Se}^{tot}$
No-vdW	0.19	3.65	3.84	0.91	1.09	2.00
D2	0.19	3.65	3.84	0.91	1.09	2.00
D3(A)	0.09	3.56	3.65	0.89	1.18	2.07
D3(B)	0.03	3.68	3.71	0.92	1.10	2.02
MLWF	0.19	3.68	3.87	0.92	1.08	2.00

**Table 2:** Structural units composing the Ge-based subnetwork of liquid GeSe_4_. The values given are the percentages of Ge atoms connected in onefold, twofold, threefold, fourfold, and fivefold fashion, the chemical nature of the different neighbors being specified in the second column.

Ge(%)		No-vdW	D2	D3(A)	D3(B)	MLWF
l = 1	Se			1.1		
l = 2	Se_2_	3.2	3.5	8.8	7.2	2.8
l = 3	Se_3_	9.4	7.9	16.3	15.5	8.3
	GeSe_2_	1.1	1.2			
l = 4	Se_4_	69.3	70.9	62.7	70.6	71.4
	GeSe_3_	11.9	12.7	6.1	2.5	11.7
	Ge_2_Se_2_	2.4	1.9			2.5
l = 5	GeSe_4_	1.1		2.6	2.9	1.3

**Table 3:** Structural units composing the Se-based subnetwork of liquid GeSe_4_. The values given are the percentages of Se atoms connected in onefold, twofold, and threefold fashion, the chemical nature of the different neighbors being specified in the second column.

Se(%)		No-vdW	D2	D3(A)	D3(B)	MLWF
l = 1	Se	3.7	3.2	4.9	5.8	3.3
	Ge	2.4	2.1	3.2	3.6	2.3
l = 2	Se_2_	29.4	30.1	27.0	26.5	29.5
	Ge_2_	23.3	23.7	18.9	22.0	23.9
	GeSe	35.0	35.5	31.9	31.5	35.6
l = 3	GeSe_2_	2.5	2.3	5.3	4.0	2.0
	Ge_2_Se	1.5	1.2	3.2	2.8	1.3
	Se_3_	1.6	1.4	3.6	2.4	1.4
	Ge_3_			1.1		

## Data Availability

Molecular dynamics trajectories are available from the authors upon request.

## Sample Availability

There are no samples, not applicable.
